# Microscale Heterogeneity of the Spatial Distribution of Organic Matter Can Promote Bacterial Biodiversity in Soils: Insights From Computer Simulations

**DOI:** 10.3389/fmicb.2018.01583

**Published:** 2018-07-27

**Authors:** Xavier Portell, Valérie Pot, Patricia Garnier, Wilfred Otten, Philippe C. Baveye

**Affiliations:** ^1^School of Water, Energy and Environment, Cranfield University, Cranfield, United Kingdom; ^2^UMR ECOSYS, Institut National de la Recherche Agronomique, AgroParisTech, Université Paris-Saclay, Paris, France

**Keywords:** soil, pore scale, organic matter, resource allocation, bacteria, biodiversity, agent-based modeling

## Abstract

There is still no satisfactory understanding of the factors that enable soil microbial populations to be as highly biodiverse as they are. The present article explores *in silico* the hypothesis that the heterogeneous distribution of soil organic matter, in addition to the spatial connectivity of the soil moisture, might account for the observed microbial biodiversity in soils. A multi-species, individual-based, pore-scale model is developed and parameterized with data from 3 *Arthrobacter* sp. strains, known to be, respectively, competitive, versatile, and poorly competitive. In the simulations, bacteria of each strain are distributed in a 3D computed tomography (CT) image of a real soil and three water saturation levels (100, 50, and 25%) and spatial heterogeneity levels (high, intermediate, and low) in the distribution of the soil organic matter are considered. High and intermediate heterogeneity levels assume, respectively, an amount of particulate organic matter (POM) distributed in a single (high heterogeneity) or in four (intermediate heterogeneity) randomly placed fragments. POM is hydrolyzed at a constant rate following a first-order kinetic, and continuously delivers dissolved organic carbon (DOC) into the liquid phase, where it is then taken up by bacteria. The low heterogeneity level assumes that the food source is available from the start as DOC. Unlike the relative abundances of the 3 strains, the total bacterial biomass and respiration are similar under the high and intermediate resource heterogeneity schemes. The key result of the simulations is that spatial heterogeneity in the distribution of organic matter influences the maintenance of bacterial biodiversity. The least competing strain, which does not reach noticeable growth for the low and intermediate spatial heterogeneities of resource distribution, can grow appreciably and even become more abundant than the other strains in the absence of direct competition, if the placement of the resource is favorable. For geodesic distances exceeding 5 mm, microbial colonies cannot grow. These conclusions are conditioned by assumptions made in the model, yet they suggest that microscale factors need to be considered to better understand the root causes of the high biodiversity of soils.

## Introduction

During the last decade, soils have become increasingly central to a number of crucial debates on issues of great societal concern (e.g., Baveye et al., 2018). Because soils contain a very large stock of carbon, there is a risk that, with rising ambient temperatures associated with global climate change, soils will release vast amounts of greenhouse gases and thereby accelerate change. Biodiversity losses have also emerged as a major concern in many parts of the world. In this context, it is not surprising that in recent years, there has been a significant surge of interest into the biodiversity of soils, and the effect it has on traditional soil functions (Nannipieri et al., 2003).

Many aspects of the biodiversity of soils have proven very difficult to understand. Soils are highly complex media in which a huge number of bacteria, archaea, and fungi live. In a single gram of soil, it is not exceptional to find as many as 10^10^ bacterial cells and 5 × 10^4^ species (Roesch et al., 2007), with commensurate numbers found for other microorganisms. To the extent that many microorganisms (an estimated 98.5% in the case of bacteria) have never been isolated or characterized, the measurement of soil biodiversity itself raises a number of fundamental questions (e.g., Nannipieri et al., 2003; Baveye et al., 2016a,b, 2018). Functionally, it is not clear at all to what extent this very large diversity of soil microbial populations is crucial and whether it needs to be preserved at all cost. Experimental results are contradictory in this respect. Whereas, e.g., Philippot et al. (2013) show that the loss of biodiversity in soils decreases denitrification activity and nitrogen cycling, the experimental results of Werts et al. (2006) suggest on the contrary that biogeochemical functions of soil such as carbon mineralization and denitrification are not impacted by a reduction of microbial diversity. Powlson et al. (2017) have recently described as an unresolved 60-year old paradox the fact that CHCl_3_ fumigation, wiping out 90% of the soil microbial population and modifying drastically its diversity, does not appear to have an effect on soil organic carbon (SOC) mineralization in soils that have a pH above 5.5. SOC mineralization continues *at the same rate*, after fumigant removal, once the initial decomposition flush is over (Powlson et al., 2017).

A similarly high uncertainty surrounds the features of soils that allow such a large microbial diversity to exist in the first place. Some researchers consider that diversity is mainly caused by biotic interactions between cells (Hanson et al., 2012), but experimental observations increasingly suggest that a high biodiversity is associated with soil spatial heterogeneity (Rainey and Travisano, 1998) and is caused by biotic and abiotic interactions taking place in the soil architecture. Yet the exact mechanisms involved remain elusive. The often advocated explanation that the heterogeneous, disconnected distribution of moisture in unsaturated soils causes distinct groups of microorganisms to be physically isolated from each other (Treves et al., 2003; Long and Or, 2009) is appealing, but it does not apply to fungi or filamentous bacteria (Baveye et al., 2016b) and cannot account by itself for the biodiversity of soils that are periodically saturated after rainfall events. At this point, there is no real, satisfactory explanation of how the spatial heterogeneity of soils might foster the biodiversity of their microbial populations.

In this general context, the key objective of the present article is to explore the hypothesis that the heterogeneous distribution of the basic nutrient resources used by bacteria in soils can account to some extent for their diversity. The spatial distribution of organic matter in soils is known to be highly heterogeneous. Incorporation of plant residues by tillage results in patchy distribution at the centimeter scale (e.g., Elyeznasni et al., 2012) while at smaller millimeter scales, heterogeneous distribution of soil organic matter has been visualized by Peth et al. (2014) and Kravchenko et al. (2014). On the basis of these microscale observations, the effect of the heterogeneity of the spatial distribution of soil organic matter and of the connectivity of the aqueous phase on bacterial biodiversity was examined in a series of scenarios using a 3D pore-scale carbon dynamics model. Bacterial cells of three strains of the *Arthrobacter sp.* including highly competitive-, generalist-, and poorly competitive-strains, were randomly placed within the 3D pore space of a small soil sample (of volume size of 314 mm^3^) imaged at a resolution of 68 μm, suitable to visualize meso- and macro-pores.

## Materials and Methods

### Soil Image

Undisturbed soil cores were sampled in the surface horizon of a cultivated soil, a silty loamy (19% clay, 75% silt, 6% sand) Albeluvisol (Vogel et al., 2015). 3D images of the samples were obtained using an X-ray CT scanner (HMX 225, NIKON metrology, Tring, United Kingdom). A global threshold according to Elyeznasni et al. (2012) was used to obtain binary images in which the voxels of the gray CT image were classified either as soil or void voxels. We selected one sub-image (called G6 in Vogel et al., 2015) of 100^3^ voxels size out of the set of segmented CT images. The voxel-resolution of the image is 68 μm, so that the pore space explored in this study encompasses most of the structural porosity made of meso- and macro-pores. This image corresponds to a volume size of about 314 mm^3^, and it has a porosity of 18.82%. This number is undoubtedly smaller than the actual porosity of the soil, because of the fact that sub-resolution pores are ignored (Baveye et al., 2017).

The localization of the fluid and gas voxels corresponding to a given water saturation index, *S_w_* (the proportion of the pore space filled with water), is calculated using a two-phase two-relaxation-times (TRT) lattice-Boltzmann model (LBM) as described by Genty and Pot (2013) and Pot et al. (2015). Three levels of water saturation of the CT-visible porosity are assumed in the present work, *S_w_* = 1.00, *S_w_* = 0.50, and *S_w_* = 0.25. After a visual inspection of the 3D distribution of the gas phase in the images, we selected a few of the smallest visible pores containing gas and recalculated from the Young-Laplace law a rough estimate of the matric potential for *S_w_* = 0.25 and *S_w_* = 0.50. The matric potentials estimated in this manner are about -0.6 and -0.3 kPa, respectively. Therefore, even though a water saturation level of 0.25 would suggest that the soil is relatively dry, the fact that this number refers only to the CT-visible pore space means that the scenarios reported in the present contribution correspond to wet conditions.

The image offers a tradeoff between resolution and sample size (constrained by X-ray computed tomography). It is a compromise that presents the advantage of reproducing the millimeter-scale variability of the microbial activity, as reported by Vieublé-Gonod et al. (2006), and of enabling us to work with a soil volume that is large enough for carbon mineralization to be measured in practice.

### Model Description

The description below of the Ib-LBioS-Comp model follows the overview, design concepts, and details (ODD) protocol (Grimm et al., 2006, 2010), which was especially developed to communicate the features of agent and individual-based models.

#### Purpose

Ib-LBioS-Comp, which stands for Individual-based LBM for biodegradation affected by soil structure and competition, is a pore scale modeling approach developed to study the impact of the soil structure or, more appropriately, architecture (see discussion in Baveye et al., 2018), molecular diffusion, spatial heterogeneity of resource distribution, and competition among bacterial species on the biodegradation dynamics of organic matter in the soil.

#### Entities, State Variables, and Scales

Ib-LBioS-Comp combines a lattice-Boltzmann solute diffusion model and an individual-based biological module describing bacterial activity. The model involves single bacterial cells (biotic agents) of up to three different species or strains, dissolved organic carbon or DOC (abiotic lattice-Boltzmann populations), and particulate organic matter (POM, abiotic agents). Bacterial cells and DOC are distributed in the 3D volume of the soil pore volume and POM is distributed in the 3D volume of the soil solid matrix. Enzymatic hydrolysis of POM is assumed to continuously supply DOC to the liquid phase. Then DOC diffuses through the liquid phase toward the microenvironments where it is taken up by bacteria and used as nutrient source. The diffusion and uptake of oxygen are not accounted for explicitly in the model at the moment and are assumed not to limit microbial activity.

The simulated space is divided into a regular 3D grid made of cubic voxels that can be either solid or filled with a fluid (air or water).

A bacterial cell or individual (*I_i_*) is defined by the variables Π*_i_*, identifying its position in the domain, its species *j_i_*, its mass *m_i_* (mg C), its specific uptake rate υ*_DOC,i_* (tu^-1^), and its mass at reproduction *m_R,i_* (mg C). All masses in Ib-LBioS-Comp are expressed in terms of mass of carbon. Letting *P* = *I*(*t*) denote the number of bacteria at time *t*, one obtains for the state of the population at time *t*:

(1) $$Pn={\left\{I_{i}\left[\Pi_{i},j_{i},m_{i},\upsilon_{DOC,i,}m_{R,i}\right]\right\}}_{i=1,2,...,n\left(t\right)}$$

where *i* is the index of individual bacteria and *n*(*t*) the total number of cells at time *t*.

A POM agent (*A_l_*) is defined by a variable indicating its position in the domain, Π*_l_*, and its carbon mass, *m_POM,l_* (mg C). Letting *A*(*t*) denote the number of POM agents, the POM population state at time *t* is:

(2) $${POM}_{m}={\left\{A_{l}\left[\Pi_{l},m_{POM,l}\right]\right\}}_{l=1,2,...,m\left(t\right)}$$

where *l* is the index of the POM agents and *m*(*t*) the total number of POM agents at time *t*.

The DOC solute is simulated by microscopic lattice-Boltzmann populations, *f_q_*, that are microscopic solute entities defined in the *Q* microscopic directions at each liquid node of the 3D grid. The *Q* directions are defined by the unit microscopic velocity vectors, $\overset{\to }{C_{q}}=\{C_{q\alpha }\}q=0,...,Q-1;\alpha =1,...,d$ where *d* is the dimension of the grid or lattice. We used the model D3Q7.

The DOC concentration in the liquid phase, *C_DOC_* (*t*) (mg C lu^-3^) can be calculated at each liquid node of the grid as the sum of the *f_q_* populations:

(3) $$C_{DOC}=\frac{1}{\Delta V_{xyz}}\overset{Q-1}{\underset{q=0}{\Sigma }}f_{q}\,$$

with Δ*V_xyz_* the volume of one voxel expressed in lu^3^ where lu is the spatial unit of the lattice, in our case determined by the scanning resolution so that 1 lu = 68 μm.

The temporal evolution of the system is divided into equal intervals associated with time steps or units (tu) of a time step length dictated by the lattice-Boltzmann submodel. The temporal extent of the simulations was set to 10 days according to previous simulations made by Vogel et al. (2015) in which exponential growth and decline of biomass were observed within this duration. The time step length is 3.44 s (see Sections “Abiotic Processes” and “Model Parameterization”).

#### Process Overview and Scheduling

**FIGURE 1:**
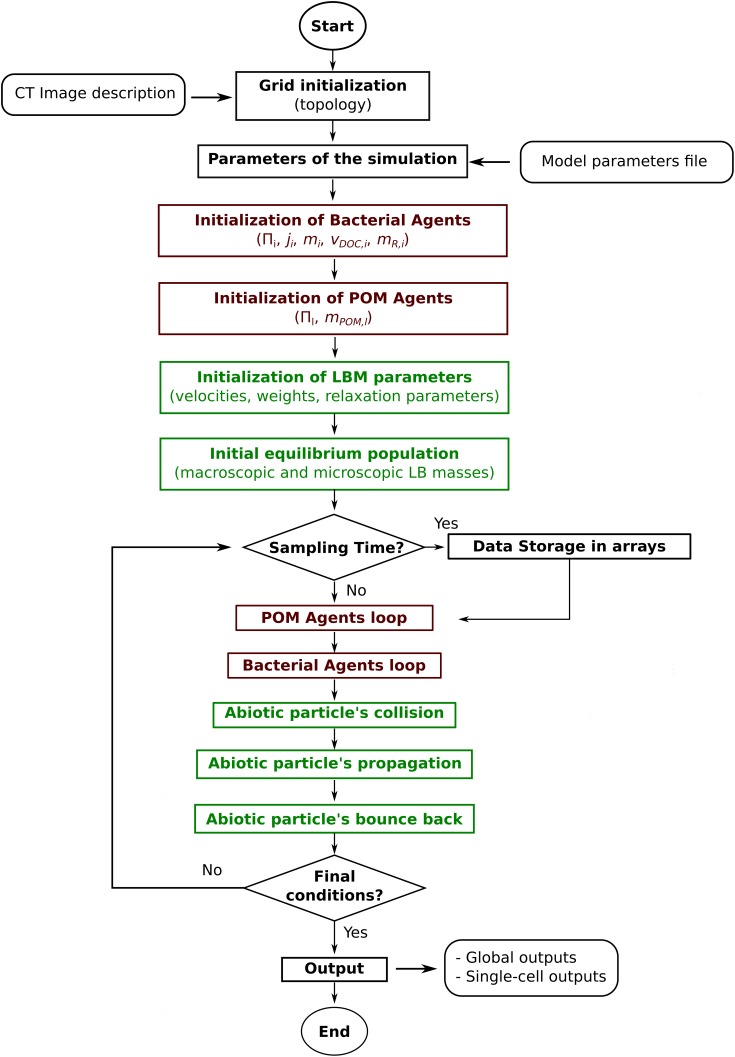
General workflow of the Ib-LBioS-Comp model.

**FIGURE 2:**
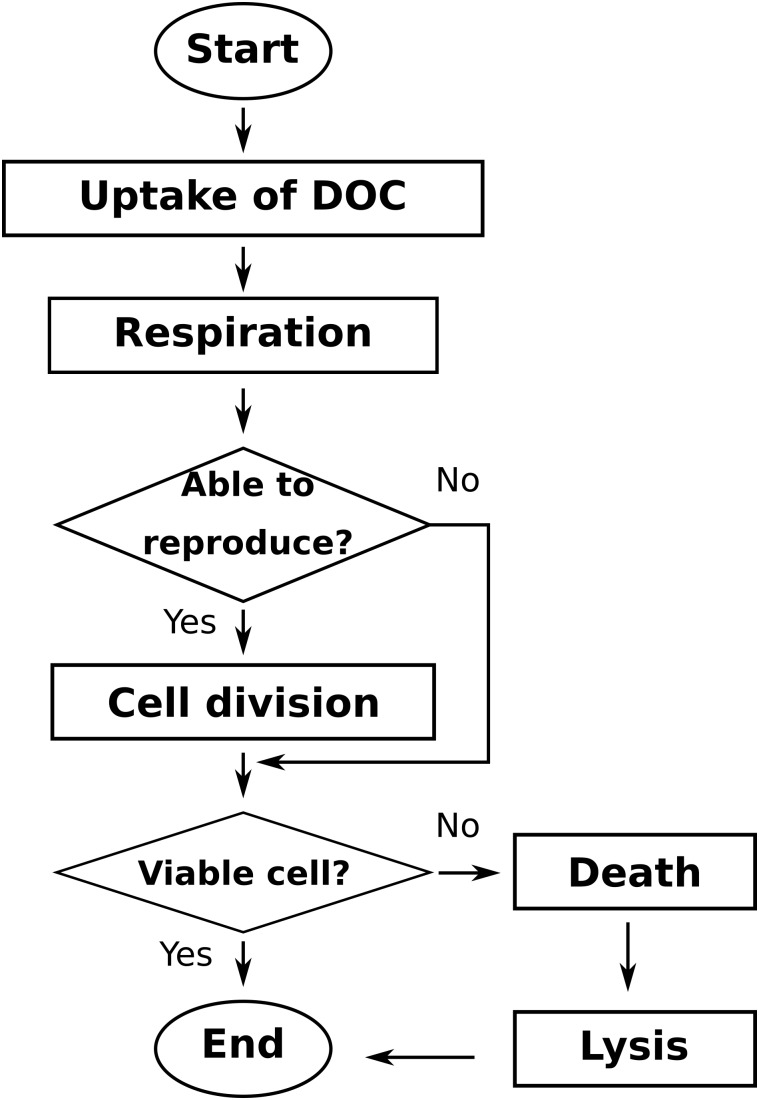
General workflow of the bacterial agents loop of the Ib-LBioS-Comp model.

Global simulation comprises three sections (**Figure 1**): (i) the initialization of the simulated system, (ii) the time step loop, which is repeated until the end of the defined time steps, and (iii) the model output section, where the system-level (aggregated) and individual-level (non-aggregated) data are saved in files. Initialization of the system includes: reading of model parameters, initialization of the bacterial agents, and initialization of the LBM parameters and DOC populations. The time step loop includes, chronologically: (ii.i) storage of the simulation state in temporary data structures, (ii.ii) the POM agents actions loop, (ii.iii) the bacterial actions loop (**Figure 2**), and (ii.iv) the lattice-Boltzmann actions. Output files of aggregated and state variables are created from the temporary data structures saved previously.

At each time step, bacterial cells perform the following set of actions: uptake, metabolism, reproduction, and mortality. The order in which bacteria act is changed randomly every time step to avoid privileging always the same first-acting bacteria. At each time step, the existing POM agents undergo hydrolysis to produce DOC. The DOC lattice-Boltzmann populations, f_q_, are then updated through the following set of actions: collision, propagation, and bounce-back when they encounter a solid or a gas neighbor. This last action is motivated by the premise that DOC occurs only in the water phase.

#### Design Concepts

##### Emergence

Bacterial processes (uptake, metabolism, reproduction, mortality) are defined at a single-cell level and the population or system level behavior emerges from the interactions among individuals and between the individuals and the media. The main population-level emerging characteristics are the population density (bacteria present in the media), the population biomass, the DOC taken up by the population, the CO_2_ produced by the population and the bacterial size distribution. Solute diffusion processes (collision, propagation) are defined at the microscopic level of the Q directions of the lattice, and the solute behavior (diffusion) emerges from the interactions among the solute lattice-Boltzmann populations.

##### Interaction

Bacteria are considered to be immobile but they compete directly for space through the maximum carrying capacity of a voxel. Therefore, the presence of other bacterial cells in the local space can directly affect the fate of new-born bacteria. Indirect interactions among individual bacteria arises from competition for the DOC available in the local environment.

##### Stochasticity

Randomness is considered when the rules are applied to individuals by using probabilistic distributions to deal with or manage individual events. Random processes or events include the assignment of the position of new-born bacteria in the physical domain near the mother when the grid element of the mother reached maximum occupancy, and the occurrence for cell death. The sequence of actions of the simulated bacteria changes randomly at each time step to avoid privileging one over the others. The model can also introduce further stochasticity when setting the mass of the initial individuals, the individual specific uptake rate, and the mass at reproduction of the individual bacteria using folded normal distributions, but this is not considered in the current study.

##### Observation

Global and single-cell outputs are recorded at the beginning of the simulation, at regular intervals during the simulation, as well as at the end of the simulation. Global variables calculated at the scale of the entire domain include: mass of DOC, POM, and CO_2_ produced in the media, and, for each of the three bacterial species, the number of bacteria, and total bacterial biomass. The state of all the individual bacteria is saved at sampling times. These single-cell data include the position within the domain, species, mass, uptake rate, and mass at reproduction of the individuals. The carbon mass and position of all the POM spots (abiotic agents) are also saved at sampling times. The DOC concentration of the liquid voxels holding at least one bacterium is also recorded at sampling times. The final DOC concentration of all the liquid grid cells of the domain is recorded at the end of the simulation.

#### Initialization

The specific uptake rate (*v_DOC,i_*), and the reproduction mass (*m_R,i_*) of the initial individuals (*i* = 4,...,*N*_*B*0_) are assumed to be specific for each *j*th species. For the individuals of each species *j*, these properties are set using the model parameters $v_{DOC}^{j}$ and $m_{t0}^{j}$, respectively, for the uptake rate and the reproduction mass. No intraspecific variability is considered in the present study. Similarly, the initial mass of the individuals starting the simulations (*m_i_*) is initialized according to the model parameters $m_{t0}^{j}$.

Since it is generally assumed that bacteria in soil microenvironments tend to be sorbed to, or be at least very near, solid surfaces, the model assumes that bacteria can be located only in liquid voxels having at least one solid neighbor. What defines a neighbor here is the particular lattice-Boltzmann connectivity that is adopted in the calculations (D3Q7 in this case). The initial *N*_*B*0_ bacterial cells are randomly distributed among a number of bacterial spots (*N*_*SP*0*T*_) that, in turn, are randomly chosen, with replacement, from the liquid voxels having one or more solid neighbors.

The POM agents are situated in the solid matrix of the soil. The initial POM agents are randomly distributed among solid voxels that have at least one liquid neighbor.

An initial amount of dissolved organic carbon, DOC_0_, is distributed homogeneously among the liquid voxels of the image.

#### Input Data

The model uses soil structural data as described in see Section Soil Image.

#### Biological Processes

Several separate submodels describe quantitatively the uptake, metabolism, reproduction, and mortality, respectively, of individual bacteria.

In the *Uptake submodel*, the uptake (*U_i_*) of carbon substrate by bacterium *i*, belonging to the species *j = j_i_*, is given by the equation depending on the mass *m_i_* (*t*) of the bacterium

(4) $$U_{i}(t)=\frac{v_{DOC,i}C_{DOC}^{\left(t\right)}}{C_{DOC}^{\left(t\right)}+k_{DOC}^{j}}m_{i}\left(t\right)$$

where *v_DOC,i_* is the specific uptake rate of the *i*th bacterium (tu^-1^), and the parameter $k_{DOC}^{j}$ is the half saturation constant (mg C lu^-3^) of the *j*th species.

In the *Metabolism submodel*, the mass of carbon taken up is used by the cell to create new biomass. Since catabolic reactions need energy, due to the respiration process, a fraction of the carbon that is taken up is released again to the media in the form of CO_2_ emissions. Accordingly, the growth of the bacterial cell is modeled using the following equation:

(5) $$m_{i}(t+1)=m_{i}\left(t\right)+U_{i}\left(t\right)-k_{r}^{j}m_{i}\left(t\right)$$

where $k_{r}^{j}$ is the respiration rate (tu^-1^) of the species *j*. Equations (4) and (5) assume that growth respiration (traditionally calculated as a function of substrate during uptake) and maintenance respiration (proportional to the biomass) are not distinguished.

*The Reproduction submodel* adopts a simple bipartition condition (Banitz et al., 2015) according to which the bacterial cell has to attain a specific individual reproduction mass before dividing. Every time step, the mass of a bacterium is compared to *m_R,i_* (mg C), the reproduction mass of the *i*th individual. If *m_i_*(*t* + 1) > *m_R,i_*, the mass of the bacterium is halved, and simultaneously a daughter cell of the same mass is created. If the number of bacteria occupying the voxel of the mother cell is less than *N_VOX_*, the maximum carrying capacity of a voxel, the cell is created in the current voxel. Otherwise, the simulator chooses randomly a voxel that has not attained the maximum carrying capacity. The searching algorithm looks progressively to neighbors situated at increasing distances (in voxels) from the mother’s voxel of origin, until all the tridimensional space is inspected. If all the voxels reach the maximum carrying capacity, the simulation stops. The daughter cell remains active but does not act until the next time step is reached. The specific uptake rate of the new-born individual (*v_DOC,i_*) and its mass at reproduction (m_R,i_) are inherited from the mother.

Finally, *the Mortality submodel* accounts for bacterial cell death derived from internal and external events (e.g., predation by other organisms). The cell cannot survive anymore due to internal events when the cell size decreases below the minimal cell size characteristic of its species *j*,$m_{MIN}^{j}$ which can be attained due to a starvation process. Cell death due to external events is accounted by a probability, $p_{M}^{j}$(dimensionless), independent of the cell state. At every time step, the submodel compares *r_p_* with $p_{M}^{j}$, where *r_p_* is a random realization coming from a uniform distribution between 0 and 1. If $r_{p}>p_{M}^{j}$ the individual bacterium dies. The cell carbon lyses and creates new DOC in the current voxel.

#### Abiotic Processes

The main abiotic processes simulated by the model are the hydrolysis of POM and the diffusion of DOC in the 3D pore space. No convective movement of DOC was considered in the model. POM agents release DOC, decreasing as a result the mass of the POM agent. The model assumes only one homogeneous fraction of POM with a unique hydrolysis rate. The hydrolysis process is modeled assuming a first order kinetics of constant rate, *k_POM_*. The underlying hypothesis is that exo-enzymes are ubiquitous in soil (Folse and Allison, 2012). Nannipieri et al. (2003) reported that newly produced exo-enzymes by bacterial cells are short-lived molecules because, for instance, proteases can degrade them. Thus, the ubiquitous enzymes are probably those physically protected though their adsorption to clay particles or humic molecules (Burns, 1982). Burns (1982) considers it a likely scenario that these enzymes become active when a POM fragment comes into contact with them. Then, the DOC released by the agent is distributed equally among the liquid voxels neighboring the solid voxels containing the POM agent.

We implemented the TRT lattice-Boltzmann approach of Ginzburg (2005). The evolution equation of the DOC microscopic entities at the liquid nodes (grid cell with k = lq), $\overset{\to }{V_{xyz}}$ from time *t* to *t*+ 1 is given by:

(6) $$f_{q}(\overset{\to }{V_{xyz}}+\overset{\to }{c_{q}},t+1)-f_{q}\left(\overset{\to }{V_{xyz}},t\right)=\lambda_{e}[f_{q}^{+}(\overset{\to }{V_{xyz}},t)-e_{q}^{+}(\overset{\to }{V_{xyz}},t)]+\lambda_{o}[f_{q}^{-}(\overset{\to }{V_{xyz}},t)-e_{q}^{-}(\overset{\to }{V_{xyz}},t)]+S_{q}$$

in which the collision and propagation steps are described, respectively, by the two first terms of right hand side and left hand side of (7), respectively. The sink/source term of DOC, *S_q_*, is calculated from the hydrolysis and bacterial processes. In the TRT scheme, the microscopic entities f_q_ are decomposed into symmetric, $f_{q}^{+}$ and antisymmetric components, $f_{q}^{-}$ along their opposite velocities $\overset{\to }{c_{q}}=-{\overset{\to }{c}}_{\overset{¯}{q}}$ (Ginzburg, 2005). During the collision step, the relaxation of moments resulting from the entities’ distribution at time *t* toward an equilibrium state $e_{q}=e_{q}^{+}+e_{q}^{-}$ governs the reorganization of the entities. The relaxation parameter λ*_e_* is a free parameter and the relaxation parameter λ*_o_* is related to the molecular diffusion coefficient, $D_{m}^{LBM}$ (lu^2^tu^-1^) along:

(7) $$D_{m}^{LBM}=\frac{-1}{3}\left(\frac{1}{2}+\frac{1}{\lambda o}\right)$$

Both parameters must be comprised between -2 and 0 for stability. The rescaling of lattice Boltzmann time units (tu) in real time units [T] is made through the relation:

(8) $$T_{R}=\frac{D_{m}^{LBM}L_{R^{2}}}{D_{m}^{R}L_{LBM}^{2}}T_{LBM}\,$$

where *T_LBM_* and *L_LBM_* are the space and time in lattice units (respectively, tu and lu) and *T_R_* and *L_R_* are their corresponding time and space units in real units [respectively (T) and (L)].

### Model Parameterization

Typical parameters for the three different strains 3R, 7R, and 9R of *Arthrobacter* sp. (see **Table 1**) were taken from the literature. The specific uptake rate, $v_{DOC}^{j}$, the half saturation constant of the uptake rate, $k_{DOC}^{j}$, and the respiration rate, $k_{r}^{j}$, of the three species were directly taken from Monga et al. (2014), while the population mortality rate, provided by the same authors, was reinterpreted as probability, $P_{m}^{j}$, dependent on the duration of the time step. These strains have different growth patterns, with 3R expected to be the fastest growing strain and strain 9R the least competitive one due to its highest value of the half saturation constant of the uptake rate. Strain 7R is supposed to be representative of a more generalist strain.

**Table 1:** Model parameters values used in the simulations. See text for an explanation of the computations and the bibliographic references used.

Symbol	Definition	Unit	Strain		
			
			*j* = 3R	*j* = 9R	*j* = 7R
**BACTERIAL PARAMETERS**
$v_{DOC}^{j}$	Maximal uptake rate	d^-1^	17	9.6	8.0
$k_{DOC}^{j}$	Half saturation constant	mg C cm^-3^ water	5.0 × 10^-4^	1.0 × 10^-3^	1.4 × 10^-4^
$k_{r}^{j}$	Respiration rate	d^-1^	0.2	0.2	0.3
$m_{R}^{j}$	Reproduction mass	mg C	1.32 × 10^-10^	1.32 × 10^-10^	1.32 × 10^-10^
$P_{m}^{j}$	Mortality probability	–	1.5	0.5	1.0
$m_{\mathrm{MIN}}^{j}$	Min bacterial mass	mg C	0.1 m_R_	0.1 m_R_	0.1 m_R_
$m_{t0}^{j}$	Initial mass of the bacterial cells	mg C	5.39 × 10^-11^	5.39 × 10^-11^	5.39 × 10^-11^
$N_{B0}^{j}$	Initial bacterial number	Cells	230	230	230

		**Unit**	**Value**
				
**BIOTIC PARAMETERS**
*N_VOX_*	Voxel Max. carrying capacity	Cells	751,423
**ABIOTIC PARAMETERS**
*k_POM_*	POM decay rate	d^-1^	0.25
*D_M_*	DOC mol. diff. coeff.	cm^2.^s^-1^	6.73 × 10^-6^


The maximum carrying capacity of a lattice-Boltzmann node, *N_VOX_*, was calculated from the volume of a single image voxel (683 μm^3^) and the mean cell volume of a bacterial cell. The mean volume of an *Arthrobacter* cell was calculated to be 0.418 μmł according to data from Erlebach et al. (2000), assuming a spherical shape for the bacterial cell. We used this mean volume for the three strains of *Arthrobacter* sp. The mean reproduction diameter (i.e., the diameter attained by the cell before division) was also estimated from *Arthrobacter* cell size distributions (Erlebach et al., 2000) and assumed to be in the range 1.25 ± 0.15 μm, which includes the biggest diameters of the size distribution measured with a Coulter counter. The central value of 1.25 μm has been used in the present study.

Then, the mean carbon content of a single cell ($m_{t0}^{j}$, **Table 1**) and the value for the reproduction mass, $m_{R}^{j}$, were calculated from the mean cell volume and the mean reproduction volume calculated earlier, assuming a density of 1.1 g/cm^3^, a ratio of dry to wet cell weight of 0.25, and a carbon content of 0.47 g C per gram of dry cells (Gras et al., 2011).

**FIGURE 3:**
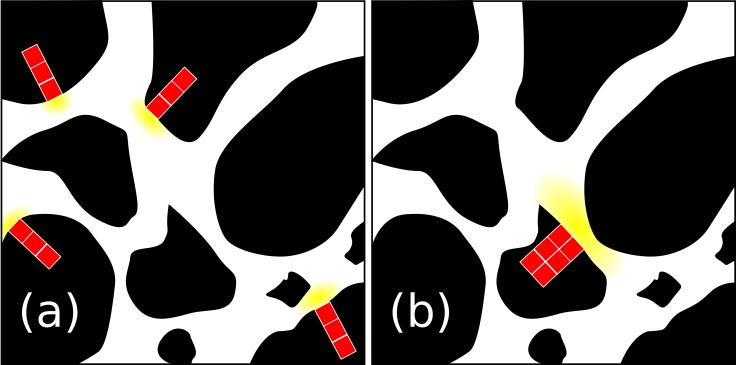
2D view of the POM initialization scheme used in the scenarios. In the figure, the POM (red), the DOC (yellow), the soil solid matrix (black), and the pore space (white) are depicted. In **(a)**, POM disaggregated, four parallelepiped POM fragments of 1 × 1 × 3 image voxels, were assumed to be present and connected to the pore space through a single voxel. In **(b)**, POM aggregated, a single 3D POM fragment of 4 × 4 × 3 was assumed to be present and connected to the pore pace through 4 of its voxels.

The value of the decay rate of the POM agents, *k_POM_*, was set to 0.25 day^-1^ as reported by Iqbal et al. (2014) for the decomposition rate of maize (*Zea mays*) stem residues. These authors found “optimal decomposition conditions similar to those obtained with ground material” for fragments of POM of 0.02 cm length. Assuming a density of POM of 0.12 g cm^-3^ (Iqbal et al., 2013) and a volume of POM residue of about 0.02 cm × 0.02 cm × 0.01 cm, we calculated an initial POM mass of carbon, POM_0_, of 1.92 10^-4^ mg C. We translated this fragment of POM into four fragments of parallelepiped shape (1 × 1 × 3 voxels), located at the solid/liquid interface (**Figure 3**). When the POM hydrolyses, the DOC produced is included in the neighboring fluid site.

The molecular diffusion coefficient in lattice-Boltzmann units, $D_{m}^{LBM}$, is fixed to 0.5 lu^2^tu^-1^ (Vogel et al., 2015, 2018). Since the DOC molecular diffusion coefficient, $D_{m}^{R}$, is 6.73 10^-6^ cm^2^s^-1^ (Weast et al., 1986) and 1 lu = 68 μm, the rescaling equation (8) gives 1 tu = 3.44 s.

### Simulation Scenarios

The scenarios were designed to investigate the effect of the local micro-environments of bacteria on the strains abundance. For all scenarios we randomly placed 230 bacteria of each strain in the medium which resulted in 690 bacterial spots. In one particular scenario (S4) the three strains systematically co-exist in the same bacterial spot, so that only 230 bacterial spots were generated. The local micro-environments of these bacterial spots were modified by introducing heterogeneity in the placement of the resource (POM and DOC) in the medium. The water content level was also modified. **Table 2** summarizes the characteristics of the different scenarios. Scenarios S1, S2, and S3 test the effect of the spatial distribution of organic matter on the global organic matter degradation and strain abundance. Scenario S4 tests the effect of the direct interspecific bacterial competition on strain abundance, whereas scenarios S5 and S6 are designed to test the effect of the water saturation level on organic matter degradation and strain abundance. Details of these scenarios are given below.

**Table 2:** Simulation scenarios overview.

Scenario	POM_0_	DOC_0_	$N_{SPOT}^{B}$	$N_{SPOT}^{POM}$	*S_w_*	Randomness
	mg C	mg C	Spots	Spots	[^-^]	
S1	1.92 × 10^-4^	0.0	690	4	0.50	POM spots
S2	1.92 × 10^-4^	0.0	690	1	0.50	POM spots
S3	0.0	1.92 × 10^-4^	690	0	0.50	Bacterial spots
S4a	1.92 × 10^-4^	0.0	230	1	0.50	–
S4b	1.92 × 10^-4^	0.0	230	1	0.50	–
S5	1.92 × 10^-4^	0.0	690	1	1.00	POM spots
S6	1.92 × 10^-4^	0.0	690	1	0.25	POM spots


*In the table: *POM*_*0*_ is the initial carbon mass of POM, *DOC*_*0*_ is the initial carbon mass of DOC, $N_{SPOT}^{B}$ is the number of bacterial spots, $N_{SPOT}^{POM}$ is the number of POM spots, and S_*w*_ is the saturation level of the media. Randomness indicates whether the POM fragments or the bacterial spots are randomly changed to generate 10 replicated simulations. The letter “a” denotes that the 230 bacterial spots are selected from the highest growing bacterial spots of repetition 2 of S2, while “b” denotes that the 230 bacterial spots occupied by the strain 9R in the repetition 2 of S2 are used.*

#### Scenario S1

The initial 690 bacteria were distributed among 690 randomly selected liquid voxels neighboring the soil solid matrix. Four POM fragments were randomly distributed in the medium (**Figure 3a**). Ten replicated simulations were performed with the position of POM fragments controlled by a random seed. The positions of the 690 bacterial spots were left unchanged for the ten replicated simulations. The water saturation level, *S_w_*, was 0.50.

#### Scenario S2

The four fragments of POM were gathered in one fragment made of 2 × 2 × 3 voxels with the base 2 × 2 voxels being contiguous solid sites neighboring 2 × 2 fluid sites (**Figure 3b**). Bacteria were located in the same 690 positions as for scenario S1 and the same water saturation level, S*_w_* = 0.50, was adopted. Ten replicated simulations were again performed with the position of POM fragments controlled by a random seed Comparison of scenario 1 and 2 made it possible to assess the effect of the degree of spatial heterogeneity of POM.

#### Scenario S3

We assumed that all the carbon that can, potentially, be hydrolyzed and released to the liquid phase of the soil in the scenarios S1 and S2, was already homogeneously distributed in the liquid phase of the medium at the beginning of the simulations as a DOC. A water saturation index of *S_w_* = 0.50 was also adopted. In ten replicated simulations, a random seed controlled the position of 690 bacterial spots. This resulted in 9 extra configurations of the 690 bacterial positions tested in S1 and S2. Comparison of S3 with S2 and S1 allowed us to evaluate the impact of homogenously- vs. heterogeneously distributed C within the soil.

#### Scenario S4

This scenario was aimed at the effect of direct interspecific competition on strain abundance. Two simulations (identified as S4a and S4b) involved 3 bacteria, one of each strain, in selected 230 bacterial spots. In S4a, we took one particular repetition of scenario S2 in which the three strains had a noticeable growth (repetition S2r2) and we classified the 690 spots according to the amount of biomass growth of the colony in a descending order. The first 230 bacterial spots were selected and used to place the initial bacteria. In S4b, we took the repetition S2r2 but we just selected the 230 bacterial positions occupied by the less competitive strain (9R). In each of these spots, we placed initially 3 bacteria, one of each strain.

#### Scenarios S5 and S6

These scenarios are identical to scenario S2 except for the water saturation level that was fixed to 1.00 (S5) and 0.25 (S6). Because the positions of the 690 bacterial spots are left unchanged, in scenario S6 there were 165 bacterial spots that were found to be in the gas phase, and thus did not grow. In the 525 spots still placed in water filled grid cells, the three strains were found to be equally distributed as 178 cells of strain 3R, 172 cells of strain 9R and 175 cells of strain 7R.

## Results and Discussion

### Effect of Spatial Distribution of Organic Matter on Global Organic Matter Degradation and Strain Abundance

**FIGURE 4:**
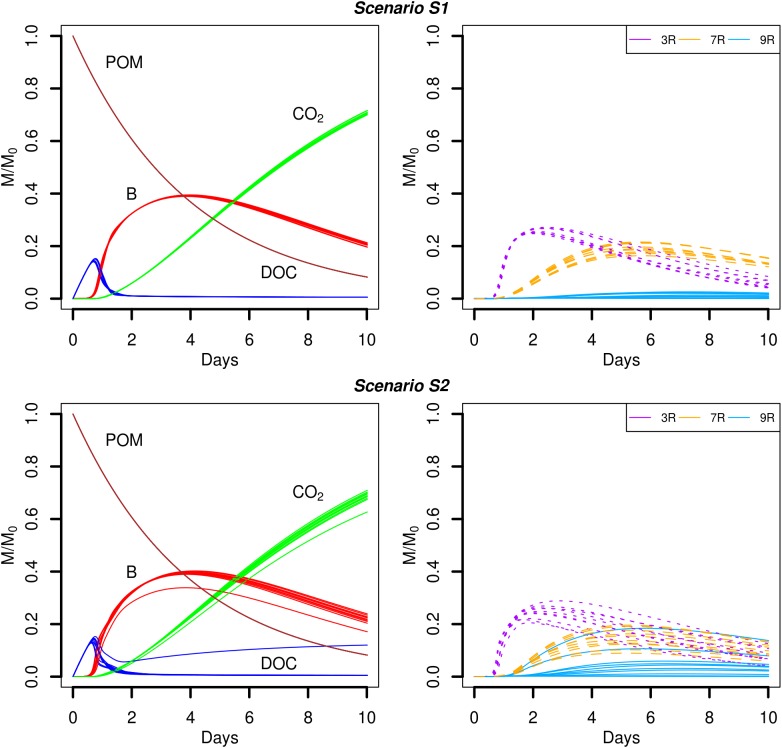
Global biodegradation kinetics (left) and strains abundance (right) of scenarios with dispersed POM fragments (S1) and aggregated POM fragments (S2). M/M_0_ is the ratio of the carbon mass (mg C) of each output over the total initial carbon mass M_0_.

Simulation scenarios S1 and S2 assume that the soil organic matter is found in a number of POM fragments that are distributed (S1) or aggregated (S2). In the proposed scenarios, the hydrolysis rate of POM is set to a constant value, so that for *k_POM_* = 0.25 d^-1^, about 92% of the initial mass of carbon of the POM fragments is hydrolyzed at the end of the simulations. The predicted time evolutions of the POM, DOC, total biomass (B), and CO_2_ are shown in **Figure 4**. At the beginning of the simulations, the carbon hydrolyzed from the POM particles cannot be totally consumed and accumulates in the liquid phase. After one day, when exponential growth of bacteria starts, the major part of the dissolved organic carbon is metabolized by the cells. At the end of the simulations, the CO_2_ emitted has not yet reached a plateau although the slope of its cumulated increase begins to decrease. Except for one repetition of scenario S2, no differences are observed in the time evolutions of the POM, DOC, total biomass, and CO_2_ between the two POM distributions.

Although the global model outputs are similar for both spatial distributions of POM, differences in the strains abundance are observed (**Figure 4**, right). When the POM is fragmented into 4 pieces, the less competitive strain 9R cannot grow significantly (**Figure 4**, scenario S1). The fastest growing strain 3R experiences an exponential growth up to two days before starting to decline. The more generalist strain 7R has a much smoother exponential increase compared to 3R, and even surpasses the biomass of 3R after five days, before starting to decline after about the day seven. When the POM fragments are gathered into a single piece, 3R has the same overall dynamics, with comparable mean abundances but with higher dispersion between replicates. The coefficient of variation (CV) of the abundance peak is 0.03 for S1 and 0.10 for S2. The strain 7R has also the same dynamics as in scenario S1 but with a lower mean abundance peak that does not exceed 78% of the 7R mean abundance peak observed for the scenario S1. Again, the CV is highest in scenario S2 compared to S1 with the values of 0.24 and 0.10, respectively. On the contrary, the strain 9R shows a much higher growth variation among replicates when the POM is aggregated in a single piece of POM. In particular, two replicates (S2r2 and S2r5) show growth kinetics similar to those of strain 7R. In three other replicates, 9R grows without exceeding the abundance of the other species. In the last five replicates, 9R presents a similar growth as in the case of POM fragmented. The S2 replicate with lower global total biomass and emitted CO_2_ (S2r9) corresponds to the lowest abundances of the strains 3R and 9R, and to the highest abundance of 7R. In that case, the highest abundance of 7R does not compensate the low abundances of strains 3R and 9R, while compensation is observed for the other replicates at the scale of the whole soil sample.

**FIGURE 5:**
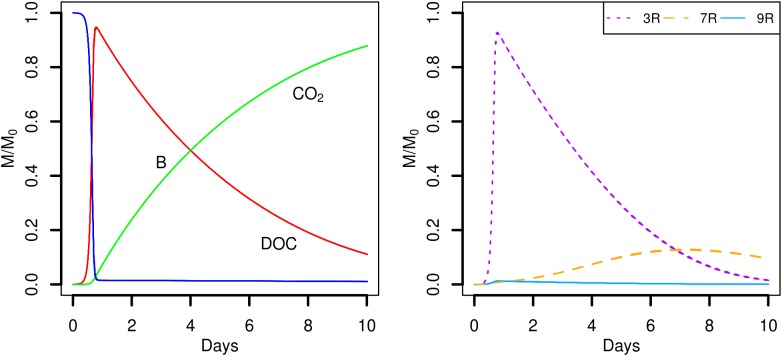
Global biodegradation kinetics (left) and strains abundance (right) of the scenario with the C available as DOC and a water saturation level of 0.5 (S3). M/M_0_ is the ratio of the carbon mass (mg C) of each output over the total initial carbon mass M_0_.

A very different biodegradation kinetics is observed when the DOC is homogeneously distributed in the pore space (**Figure 5**, scenario S3). The global growth of the total biomass is much faster (**Figure 5**, left) with a maximum peak reached at day one. The value of the peak is 4.5 times higher than the peaks reached by the total biomass in scenarios with POM fragments. The higher amount of DOC available initially permits a higher bacterial uptake that translates into a faster growth of the bacteria. As a result, the amount of DOC quickly decreases within the same time interval (one day) and the cumulated CO_2_ emitted is higher, with values of about 72% (**Figure 4**) and 91% (**Figure 5**), respectively. The different picture shown by the biodegradation kinetics has relevant implications for the species abundance (**Figure 5**, right). The most competitive 3R strain dominates from the very beginning. The high value of its maximal uptake rate (**Table 1**) makes 3R cells benefit more from the larger initial DOC concentration, and experience a quick and large exponential growth. This strain shows a more pronounced decline phase than in scenarios with POM fragments. Only when DOC becomes scarcer, the more generalist strain 7R with the lowest *k_DOC_* values succeeds to grow at about the same extent as observed in previous scenarios with POM fragments. The strain 9R with intermediate growth rate but with a higher *k_DOC_* value is not competitive enough to grow. A probable explanation is that, after two days, the DOC concentration at the local microbial habitat of strain 9R remains always lower than in the case of the POM fragments. In the latter case, some microbial habitats of strain 9R that are located close to the POM fragments can still benefit from a sufficient DOC concentration for them to grow. On the contrary, the more generalist strain 7R is not impacted by the lower DOC concentrations. **Figure 5** also shows that the position of the bacterial spots does not affect the predicted model outputs, and the ten replicated simulations almost overlap. Simulation scenario 3 is consistent with the nutritional state of the soil after a sudden flush of nutrients, which is typical of the anthropic addition of fertilizers to agricultural land, or as observed after rainfall following a period of drought.

### Effect of Spatial Distribution of Organic Matter on Spatial Distribution of Strain Abundance

**FIGURE 6:**
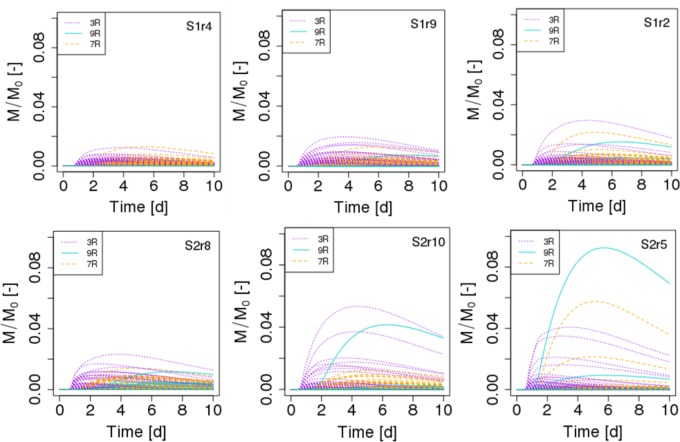
Biomass growth kinetics of the three strains (3R, 9R, and 7R) of each of the 690 spots in scenarios of dispersed POM fragments (S1, Top figures) and aggregated POM fragments (S2, Bottom figures). M/M_0_ is the ratio of the carbon mass (mg C) of the cells over the total initial carbon mass M_0_. For each scenario, the replicate having the minimal, mean and maximum biomass growth is displayed from left to right.

The spatial distribution of the abundances of the different strains in the scenarios S1 and S2 reveals a number of interesting patterns. In terms of the biomass in each of the 690 spots, we observe that the maximum peak abundance reached in the spots is 2.43 ± 1.01 higher in the case of scenario S2 compared to scenario S1 (**Figure 6**). In both scenarios, a few spots containing cells of the less competitive strain 9R can surpass spots of strain 3R and 7R and even be the most active spots (simulations S2r5 **Figure 6**, and simulation S2r2, not shown). When POM is fragmented, some spots containing 9R cells also end up with an amount of biomass that is similar to what is found with the strains 3R and 7R (simulations S1r2 and, to a lesser extent, simulation S1r9, **Figure 6**) showing that the global, per strain representation displayed in the graphic at right in **Figure 4** hides the very large dispersion of kinetics at the local scale (that of the microbial habitat).

A closer look at the microbial habitats suggests that 10 ± 1% of the spots do not experience any bacterial growth in scenarios S1. Among those spots, about 45% contain one initial bacterial cell of strain 3R, while 21% contain one initial 9R cell, and 34% contain one initial 7R cell. A higher proportion of non-active spots is found in scenario S2, 18.5 ± 5%. A similar distribution of strains among those spots is observed for the scenario S1. When it comes to the active spots, when POM is fragmented in four pieces, only at 9.5 ± 4.0% of the spots does biomass exceed 10% of the maximum biomass registered among all the spots. Among these spots, about 60% contain 3R cells, 38% contain 7R cells and only 2% contain 9R cells. When POM is present in a single piece, the proportion of active spots exceeding 10% of the maximum simulated biomass, drops to 4.2 ± 2.4%. Among those spots, about 58% contain 3R cells, 33% contain 7R cells, and 8% contain 9R cells. The higher proportion of 9R cells spots among the most active spots in scenario S2 compared to scenario S1 explains the observed higher abundance of this strain.

The general trend suggested by these results is that when POM is gathered into one piece, significantly fewer microbial habitats (almost half) are prone to grow, and a larger dispersion of the abundance is found compared to scenarios in which POM is fragmented. Therefore, under these conditions, it is not surprising that similar biomass growth is observed at the scale of a soil sample (**Figure 4**, left). In our scenarios, since the POM hydrolysis is constant and independent of the spatial position of bacteria, a lower amount of dissolved organic carbon is produced locally compared to the aggregated POM fragments when there are 4 fragments. Even if the probability of having more spots closer to these local sources of DOC is higher with dispersed POM, the available DOC concentration remains lower. Gaillard et al. (1999) show that an aggregated distribution of organic matter mineralized a lower amount of carbon than a dispersed distribution. They explain their results on the basis of a higher exchange surface with soil of the dispersed distribution. The simulation scenarios performed in this contribution were designated to create a similar contact surface area between the soil solid and liquid phases and, therefore, cannot account for the reported outputs. Nevertheless, in general, differences in the contact area still constitute a plausible explanation for differences in mineralization rate in soils.

**FIGURE 7:**
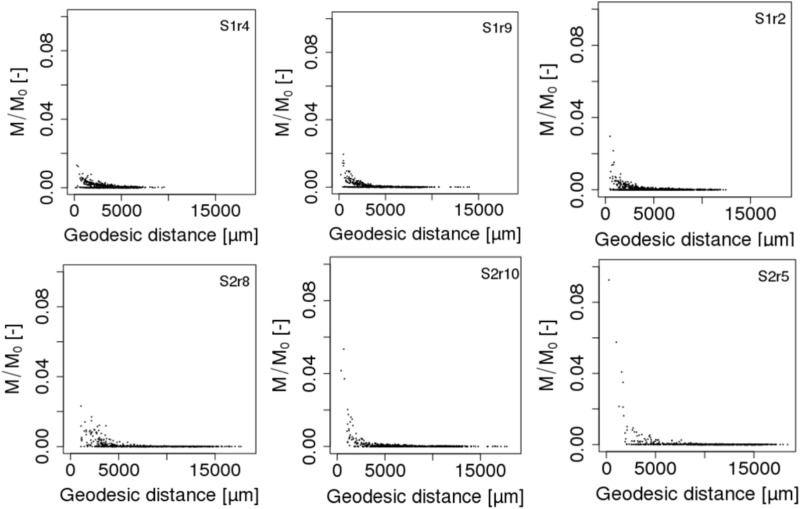
Total biomass growth of each of the 690 spots against the geodesic distances between the spots and the POM fragments of scenarios S1 (Top figures) and S2 (Bottom figures). M/M_0_ is the ratio of the carbon mass (mg C) of the cells over the total initial carbon mass M_0_. For each scenario, the replicate having the minimal, mean and maximum biomass growth is displayed from left to right.

Using an algorithm developed by Dijkstra (1971) and based on the 6-connexity of the lattice-Boltzmann grid, we further calculated the geodesic distance between each of the 690 spots and the POM fragments in order to relate biomass growth of the microbial habitats to their spatial remoteness of POM. The geodesic distance is the shortest pathway included in the liquid phase that connects two points in the pore space (Gommes et al., 2009). Divided by the Euclidian distance, the direct pathway between the two points, it gives the geometrical tortuosity as defined by Clenell (1997). Geometrical tortuosity and constriction are often considered to be good descriptors of the diffusive transport of solutes in complex pore spaces (Berg and Held, 2016). The mean geodesic distances of the bacterial spots to the POM fragments are about 1.6 times longer when POM fragments are gathered with 5,041 ± 3,190 μm and 8,144 ± 3,983 μm for scenarios S1 and S2, respectively. The mean smallest geodesic distances are 252 ± 111 μm and 490 ± 262 μm for S1 and S2, respectively. Within one replicate of either scenario, the most active spots are those having the shortest geodesic distance (**Figure 7**), however, when comparing the repetitions within a scenario it appears that spots where growth is maximum (S1r2) has a longer or similar minimal geodesic distance than spots having lower growth (S1r9 and S1r4). A large number of spots that do not have significant growth can have a very large range of geodesic distance values (**Figure 7**), which is an unexpected result. In some cases, the geodesic distance can even be close to the shortest values. However, when geodesic distances are longer than about 5000 μm in the case of S1 and about 7500 μm in the case of S2, the spots do not noticeably grow. When POM fragments are dispersed, the geodesic distances are shorter but also the DOC concentration that is emitted from the POM spots is lower.

To our knowledge, it is the first time that geodesic distances between the nutrient resource and microbial habitats are calculated in 3D modeling scenarios of soil carbon dynamics. They reveal that beyond a distance of 5 mm to the POM fragments, the microbial colonies cannot grow. Interestingly, Euclidian distances of around 4 mm from straw labeled with ^13^C have been reported to hold the sites of higher microbial assimilation and referred as “residusphere” (Gaillard et al., 1999). However, although the most active microbial spots are correlated with the lowest geodesic distances, a low geodesic distance is not a sufficient condition for the microbial colony to grow. We suggest that the size of the pores also matters (e.g., discussion in Baveye et al., 2018). Large cavities can dilute the concentration of DOC that reaches the bacteria, impacting ultimately the growth in the microbial habitat. Therefore, a bacterial spot can experience a microscale environment promoting more the bacterial growth than a spot placed at a shorter geodesic distance to the POM. Calculation of constriction in addition to diffusion length would be more appropriate. More effort is thus needed to calculate other metrics of importance for diffusive transport such as the constriction factor and the diffusion length in order to characterize and fully understand species abundance and functioning at pore scale.

### Effect of Direct Interspecific Competition on Strain Abundance

**FIGURE 8:**
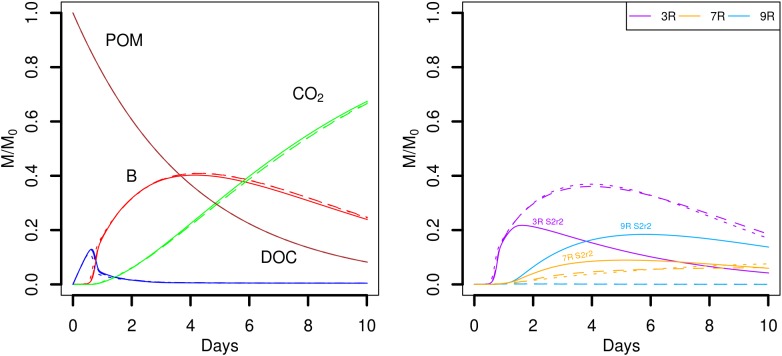
Global biodegradation kinetics (left) and strains abundance (right) of replicate 2 of the scenario with aggregated POM fragments (S4) in which the three strains are initially alone in the spots (S2r2, solid lines) or gathered in the spots (S4a dotted lines and S4b dashed lines). M/M_0_ is the ratio of the carbon mass (mg C) of each output over the total initial carbon mass M_0_. Simulation outputs obtained in the second repetition of the scenario S2 (solid lines) are compared to the two simulations of the scenario S4 (dashed lines).

Simulation scenarios S1, S2, and S3 suggest that when the strain 9R is closely competing with 3R or 7R, growth of 9R can occur only when it is located in an advantageous point with respect to a sufficient source of DOC (for instance in the case of gathered POM fragments, **Figure 4**, scenario S2). To test this hypothesis, we chose one replicate of the scenario S2 (S2r2) in which a few 9R microbial habitats experienced growth, and we initially placed three cells in now 230 spots, one of each strains. The global outputs of the model are similar (**Figure 8**, left), but the strain abundances are very different (**Figure 8**, right). When the three strains co-exist in the same microbial habitat, the strain 9R cannot grow, even when it is located in the spots close to the POM fragments. The most competitive strain 3R grows to a much higher extent and the strain 7R has a delayed growth. There are no prominent differences observed between scenario S4a and S4b.

### Effect of Water Saturation Level on Organic Matter Degradation and Strain Abundance

**FIGURE 9:**
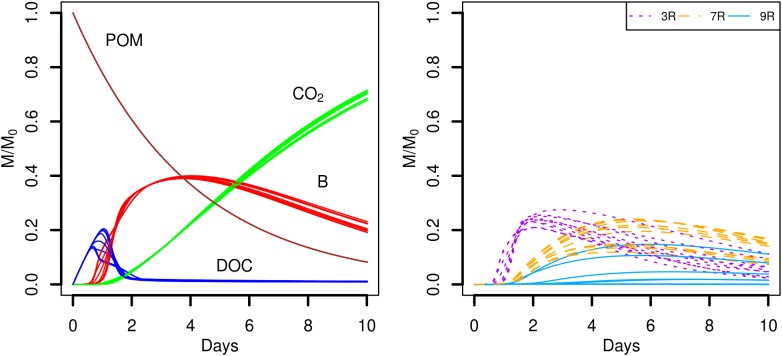
Global biodegradation kinetics (left) and strains abundance (right) of the scenario with aggregated POM fragments and a water saturation level of 1.0 (S5). M/M_0_ is the ratio of the carbon mass (mg C) of each output over the total initial carbon mass M_0_.

The role of the water saturation level of the pore space on the carbon dynamics was investigated in the scenarios S5 and S6. The global model outputs are similar when the pore space is fully saturated with water (*S_w_* = 1.00, **Figure 9**, scenario S5). As mentioned in Section Entities, State Variables, and Scales, oxygen limitations are not yet considered in Ib-LBioS-Comp, so that the different water saturation levels do not impact the role of oxygen in the bacterial activity in these scenarios. Furthermore matric potentials considered in the scenarios S2 and S6 are very close (about -0.3 and -0.6 kPa, respectively) so that oxygen limitations are not expected to happen under the tested conditions. Since the local positions of the aggregated POM fragments and the 690 microbial habitats are not changed, the only effect of the higher water saturation is to decrease the DOC concentrations. However, the diffusive transport of DOC is also accelerated because all the pore space is now connected. The strain abundances are rather similar although this environment is comparatively more favorable for the generalist strain 7R, which becomes the most abundant strain after day 6. The establishment on the system for the simulations showing a noticeable growth of the less competing strain appear mostly driven by the spatial heterogeneity of POM distribution.

**FIGURE 10:**
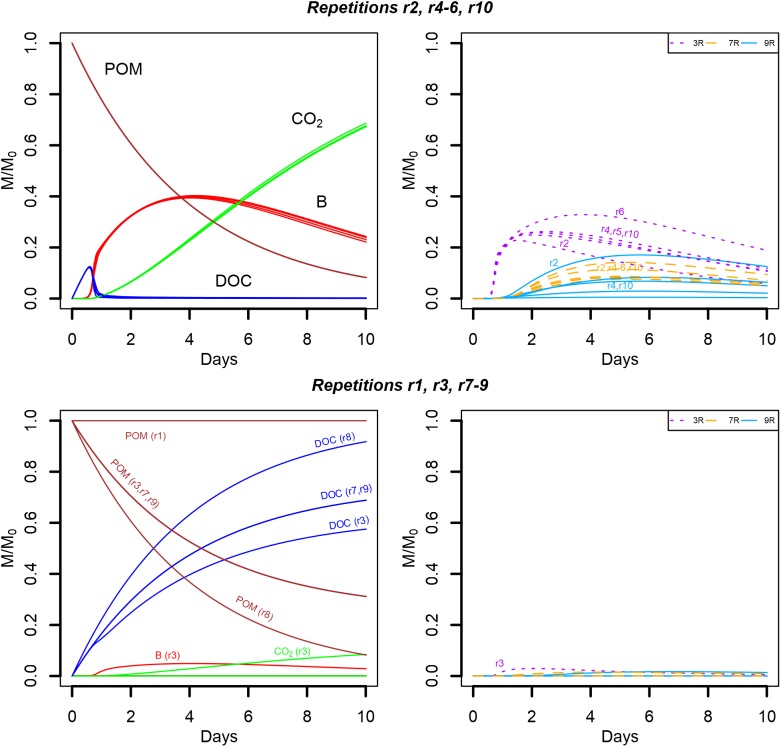
Global biodegradation kinetics (left) and strains abundance (right) of the scenario with aggregated POM fragments and a water saturation level of 0.25 (S6). M/M_0_ is the ratio of the carbon mass (mg C) of each output over the total initial carbon mass M_0_. The repetition number generating the trends depicted are detailed in the figure.

When the water saturation level decreases, the pore space filled with air increases and more disconnected aqueous regions appear. Consequently, some POM spots may not hydrolyze resource to the liquid phase, and, some bacterial microhabitats may not have access to the DOC hydrolyzed by the connected POM agents. In particular, when the water saturation is divided by two (from *S_w_* = 0.50 to *S_w_* = 0.25), 525 of the original bacterial spots are still in the aqueous phase. In spite of that, five of the replicates (S6r2, S6r4, S6r5, S6r6, and S6r10) produced global model outputs similar to the ones observed for higher water saturation levels (**Figure 10**, upper panel). In one replicate (S6r1), the aggregated POM fragments are located in solid voxels whose neighbors are disconnected from the aqueous phase, preventing the release of DOC, and thus bacterial growth (**Figure 10**, lower panel). In three other replicates (S6r3, S6r7, and S6r9) one of the four gathered solid voxels containing the aggregated POM fragments also has a dry neighbor voxel. Furthermore, for two of them (S6r7 and S6r9), and for the replicate S6r8, no bacterial growth is recorded, resulting in the accumulation of DOC in the liquid phase (**Figure 10**, bottom left). In these three last situations, spatial disconnections between the microbial spots and the POM fragments are made possible by the aqueous phase fragmentation. Spatial disconnections are also observed in the repetition S6r3 where total biomass growth is very small (**Figure 10**, bottom). The strain abundances in the active spots are very similar to those observed when the water saturation is 0.50. In one repetition, the growth of strain 9R of all microbial spots surpasses the growth of strain 7R (**Figure 10**, upper right).

It is known that lack of competition resulting from spatial disconnection of soil microhabitats may promote biodiversity (Kim et al., 2008; Vos et al., 2013). Complete or partial spatial separation due to disconnection of liquid soil volumes in unsaturated soils is a common hypothesis used to explain biodiversity. For instance, one study using experimental setups with two bacterial strains competing for a dissolved resource in sand showed dominance of the more competitive strain under water-saturated conditions while drier conditions allowed the less competitive bacteria to establish (Treves et al., 2003). Regardless of the high variability on the model outputs found for the drier conditions (*S_w_* = 0.25), our scenarios do not show a clearly improved establishment of the less competing strain. Another study (Zhou et al., 2002), based on an rRNA-based cloning approach, reported differing biodiversity distributions in the microbial communities living in four geographically distinct sites at different soil depths. A uniform biodiversity distribution, which is thought to arise from a lack of microbial competition, was obtained for the saturated subsurface of both high and low carbon soils. Since the hypothesis of resource disconnection is difficult to hold under water-saturated conditions, the authors explain their observed pattern by a lack of competition due to specialization for different substrates. Although this hypothesis remains plausible, our simulations suggest that the spatial heterogeneity of the resource placement could also explain part of this biodiversity.

## Conclusion

We have coupled a multi-species individual-based model describing bacterial growth to a 3D lattice-Boltzmann diffusion model to simulate organic matter dynamics in soil pore space. The resulting model, Ib-LBioS-Comp, has been used to study the influence of the spatial heterogeneity of a nutrient source on the organic matter degradation and species abundance of a competitive-, a generalist-, and a poorly-competitive bacterial strain. The scenarios used three resource placements showing a gradient in the spatial heterogeneity of its distribution: organic matter dissolved in the aqueous phase (low heterogeneity), four fragments of organic matter (intermediate heterogeneity), and a single fragment of organic matter (high heterogeneity).

A number of results can be highlighted from the modeling scenarios performed: (i) In general terms, the greater the spatial heterogeneity of the location of the resource, the greater the variability in the output at the level of the soil volume imaged; (ii) When the resource is found as particulate organic matter, the fastest growing strain tends to dominate at first but then, when the resources becomes scarcer, it is overtaken by the generalist strain, showing that the spatial distribution of organic matter affects bacterial succession; (iii) the global bacterial growth is faster when the nutrient resource is available in the liquid phase at the beginning of the simulation. Under these circumstances, the fastest growing strain is able to reach much higher relative abundances, having a negative effect on biodiversity; (iv) When the resource is present as particulate organic matter, the total biomass created does not differ noticeably between the intermediate and high spatial heterogeneity schemes but in contrast the species abundance is impacted; (v) The least competing strain, which does not reach noticeable growth for the low and intermediate resource spatial heterogeneity schemes, is able to grow appreciably in the absence of direct competition, if the position of the nutrient resource is favorable. According to this observation, heterogeneity of the spatial distribution of the organic matter in soil would promote microbial diversity; (vi) the geodesic distance among the nutrient resources and the bacteria alone is not sufficient to explain this phenomenon; and, (vii) In the scenarios tested, the water saturation level does not seem to change much the observed biodiversity.

As a cautionary note, one needs to remember that the various predictions made in this article emanate from a relatively simple model, which ignores many different aspects of soils. In actual soils, microbial diversity and growth are potentially affected by a myriad of factors (e.g., resource quality, different nutritional requirements of individual bacteria, mutation, predation, transport by soil fauna, toxin production). In particular, bacterial motility and the diffusion of oxygen in the pore space are likely to have a significant influence on microbial activity. Subresolution pores, too small to be visible in X-ray CT images, are most probably playing a role in this context as well, especially in the dry range of the hydrological regime of soils. Future research will determine if the relationship between bacterial diversity and the spatial heterogeneity in resource distribution, highlighted in the present article, still holds when some of the assumptions of the model are lifted. It is possible that the same relationship will be observed, or that other, more complex behaviors will unfold.

## Data Availability

Data underlying this paper can be accessed at https://doi.org/10.17862/cranfield.rd.6744158.

## Author Contributions

XP implemented the models. XP and VP ran simulations and analyzed the data. All authors conceived, designed the study, and contributed to the writing of the manuscript.

## Conflict of Interest Statement

The authors declare that the research was conducted in the absence of any commercial or financial relationships that could be construed as a potential conflict of interest.
